# The function role of ubiquitin proteasome pathway in the ER stress-induced AECII apoptosis during hyperoxia exposure

**DOI:** 10.1186/s12890-021-01751-9

**Published:** 2021-11-22

**Authors:** Yue Zhu, Huimin Ju, Hongyan Lu, Wei Tang, Junying Lu, Qiuxia Wang

**Affiliations:** Department of Pediatrics, Affiliated Hospital of Jiangsu University, Zhenjiang, Jiangsu 212000 People’s Republic of China

**Keywords:** Bronchopulmonary dysplasia, Endoplasmic reticulum stress, Ubiquitin proteasome pathway, Alveolar epithelial type II cells, Apoptosis

## Abstract

**Background:**

Bronchopulmonary dysplasia (BPD) is a major cause of mortality and morbidity in premature infants, characterized by alveolar dysplasia and pulmonary microvascular remodeling. In the present study, we have investigated the functional roles of ubiquitin proteasome pathway (UPP) in BPD, and its relationship with endoplasmic reticulum stress (ERS) mediated type II alveolar epithelial cell (AECII) apoptosis.

**Methods:**

A hyperoxia-induced BPD rat model was constructed and the pathologic changes of lung tissues were evaluated by hematoxylin–eosin staining. Cell apoptosis and protein expression were determined by TUNEL assay and Western blotting, respectively. Further reagent kit with specific fluorescent substrate was utilized to measure the activity of 20 s proteasome. Meanwhile, AECII were cultured in vitro and exposed to hyperoxia. AECII apoptosis were measured by flow cytometry. In contrast, MG132 treatment was induced to explore UPP during hyperoxia exposure on AECII apoptosis and ERS sensors expression.

**Results:**

A significant increase in apoptosis and total ubiquitinated proteins expression were observed in BPD rats and AECII culture, and the change of UPP was associated with ERS. In order to confirm the role of UPP in AECII apoptosis of BPD, AECII cells were treated by MG132 with the concentration of 10 μmol/L under hyperoxia exposure. We found that the proteins expression of glucose-regulated protein 78 (GRP-78), PKR-like ER kinase (PERK), activating transcription factor 4 (ATF4), activating transcription factor 6 (ATF6) and C/EBP homologous protein (CHOP), as well as AECII apoptosis were increased following MG132 treatment. Furthermore, the relatively up-regulated in the levels of total ubiquitinated proteins expression and 20 s proteasome activity were correlated with increased ERS sensors expression.

**Conclusions:**

Our findings indicate that UPP may participate in the ERS-induced AECII apoptosis under hyperoxia condition.

**Supplementary Information:**

The online version contains supplementary material available at 10.1186/s12890-021-01751-9.

## Introduction

Bronchopulmonary dysplasia (BPD) was defined as the need for mechanical ventilation and oxygen supplementation at 28 days of life and at 36 weeks of gestation [1]. Type II alveolar epithelial cell (AECII) is the main stem cells in the lung, which maintains normal pulmonary function. Previous reports have shown that hyperoxia induce excessive apoptosis in AECII, which play an important role of the BPD development [2–4]. AECII is the place for pulmonary surfactant protein (SP) synthesis and secretion, the cytoplasm contains large number of lamellar body and rough endoplasmic reticulum (ER). Prolonged exposure to hyperoxia results in the accumulation of misfolded proteins in the ER, and activation of unfolded protein response (UPR) [4–8]. However, a precise mechanism linking AECII apoptosis to BPD is not entirely understood.

UPR is an ER protective mechanism that restores the ER integrity under cellular stress conditions. UPR involves the activation of three ER proteins: PKR-like ER kinase (PERK), Inositol-requiring enzyme 1(IRE1), and activating transcription factor 6 (ATF6). UPR activation plays two major roles: 1) suppression of most protein translations through phosphorylation of eukaryotic translation initiation factor 2 subunitα (eIF2α) by PERK; and 2) over-expression of ER chaperones (GRP78, GRP94 and heat shock proteins), proteins involved in protein folding and components of ER-associated degradation. Prolonged ER stress activates an ER stress-dependent apoptotic pathway by induction of C/EBP homologous protein (CHOP), also known as GADD153 and DDIT3 protein [9–12]. Our previous study found that cell apoptosis was increased, and the expression of GRP78, activating transcription factor 4 (ATF4) and CHOP were up-regulated in hyperoxia exposed neonatal rats, suggesting that ERS related apoptosis pathway is involved in hyperoxia induced lung injury [13].

Under the monitoring of endoplasmic reticulum, unfolded or misfolded proteins are specifically recognized, transported to the cytoplasm, and then degraded by ubiquitin proteasome pathway (UPP), which is called ER associated degradation (ERAD) [14]. When the proteasome activity is inhibited, some proteins or transcription factors with regulatory functions could not be smoothly degraded, which will break the balance of the protein system and even start the apoptosis process. Previous research showed that in the early stage of hyperoxia exposure, UPR is activated in rat lung tissue [15]. It is worth mentioning that ubiquitin protein expression is increased in AECII and bronchial epithelial cells during hyperoxia condition, especially in AECII [16]. Nguyen et al. [17] found that proteasome inhibitor MG132 (z-leu-leu-cho, tripeptidyl acetaldehyde) can induce ERS in alveolar epithelial cells, in which the three receptor proteins of UPR are activated, and the cells tend to apoptosis.

However, it is unclear whether UPP and ERS are related to imbalance of AECII apoptosis in BPD. Here, we explored the functional roles of ERS and its association between apoptosis, thus confirmed the regulatory effect of UPP in the ERS mediated AECII apoptosis by using proteasome inhibitor MG132 in AECII during hyperoxia condition.

## Materials and methods

### Animals and tissue preparation

Sprague–Dawley rats (SD, 90–100 days old, 250–300 g) were provided by the Animal Center of the Jiangsu University (Zhenjiang, China). The BPD animal model was constructed as previously described [18]. Newborn SD rats were randomly divided into two groups, and were exposed to hyperoxia (80–85% O_2_; hyperoxia group) and room air (21% O_2_; normoxia group) at the beginning of the day of their birth. Three newborn rats from different litters per group were dissected and their lungs were removed at the postnatal day 7 and day 14 (P7 and P14). The left lungs were fixed with 4% paraformaldehyde and the right lungs were stored at − 80 ˚C.

### Histological analysis

Tissues were fixed with 4% paraformaldehyde for 24 h at 4˚C and washed with PBS. Subsequently, samples were dehydrated using an alcohol gradient (75% alcohol, 1.5 h; 95% alcohol, 1.5 h; 100% alcohol, 1.5 h; 100% alcohol, 1 h; two xylene washes, 0.5 h each) and embedded in paraffin. Sections were sliced at 3 µm, followed by conventional dewaxing in water. Antigen retrieval was performed in 10 mM citrate buffer (pH 6.0) and boiled for 20 min. The tissue sections were stained with hematoxylin and eosin (Solarbio Science & Technology Co., Ltd., China) 2–3 min each for histological analysis. All steps were performed at a room temperature. The sections were acquired by confocal light microscopy (Olympus Corporation, Japan) at magnification of × 400. Radial alveolar counts (RACs), representing alveolar septation and alveologenesis, were determined by standard morphometric techniques [19]. From the center of the respiratory bronchiole, a perpendicular was drawn to the edge of the acinus, defined by a connective tissue septum or the pleura, and the number of septa intersected by this line were counted.

### TUNEL analysis for lung tissue

TUNEL assay was applied to detect the apoptotic cells in lung tissue samples. Lung tissues were fixed with 4% paraformaldehyde, embedded in paraffin wax, and sectioned into 4 μm thick slices. The tissue section was then dewaxed and rehydrated. A Dead End Fluorometric TUNEL System (Vazyme Biotech Co., Ltd., China) was used to perform the TUNEL assay on the isolated lung tissue, following the manufacturer’s instructions. Finally, 5 random fields were selected in each section and the TUNEL-positive cells were calculated by an inverted fluorescent microscope at magnification of × 400. The apoptosis index was determined as the percentage of the total cells positive for TUNEL.

### AECII isolation and culture

At the gestational age of 19–20 days, the lung of fetal rat was in the small tubular phase. In this period, AECII produce a large number of lamellar bodies and secrete large amounts of alveolar surfactant. The structure and functional characteristics of human fetal lung at 34–35 weeks are similar with that of prenatal day 19–20. So we selected 19 fetal rat for this study. Isolation and culture of fetal AECIIs was performed as previously described [20, 21]. In brief, lungs of 19-day gestation fetal rats (Term 22 days) were removed, dissected free from connective and nonparenchymal pulmonary tissues. Cells were dispersed using a solution of trypsin, Dnase and collagenase. AECIIs were extracted from a cell suspension utilizing a property of fibroblasts and other lung cells to adhere to plastic. Freshly isolated AECIIs were plated at 5 × 10^5^ cells/mL in 50 ml culture flasks in 2 ml of MEM containing 10% fetal bovine serum. The cells were incubated for 18–20 h at 37 °C in 5% CO_2_ atmosphere. AECIIs were characterized by their morphologic appearance and the presence of lamellar bodies. All cultures contained 94 ± 2% (mean ± SE) AECIIs as determined under phase contrast microscope.

### Cell grouping and MG132 treatment

AECIIs were divided into two groups according to the conditions of their maintenance, “normoxia group” and “hyperoxia group”. The normoxia group was cultured in 5% CO_2_ incubator at 37 oC.The hyperoxia group was exposed to the stream of 95% O_2_ and 5% CO_2_ at a speed of 3 l/min for 10 min, then sealed and cultured in a parallel with normoxia group in 5% CO_2_ incubator for 24, 48 and 72 h at 37° C. To explore ubiquitin proteasome pathway during hyperoxia exposure, AECII cells were divided into Control group (hyperoxia group), DMSO group (hyperoxia + DMSO) and MG132 group (hyperoxia + MG132). MG132 was purchased from MCE (MedChemExpress, USA) and dissolved in dimethyl sulfoxide (DMSO). The method of hyperoxia exposure is the same as before. The oxygen concentration in the cells of hyperoxia group was detected by CYS-1 digital oxygen monitor when the cells were harvested. The samples with oxygen concentration less than 90% were discarded and the remaining cells were harvested for the next experiment.

### Cell viability assay

AECII cells were treated by MG132 with varying concentration (0, 5, 10, 15 and 50 μmol/L) and cell viability was detected by MTT assay. The MTT assay involves the reduction of the soluble yellow dye (MTT) to an insoluble purple formazan salt. Cells were cultured in a sterile 96 wells plate in 100 μl media and incubated overnight for attachment. AECII cells were treated by MG132 for 24 h, and at the end of reaction 50 μl of MTT dye (5 mg/ml) was added to each well and incubated further for 4 h at 37 °C in a CO_2_ incubator. The formazan products formed in cells were dissolved in DMSO (100 µl) and absorbance was measured at 540 nm using multimode plate reader (Perkin Elmer, USA).

### AECIIs apoptosis assay

The cells were treated with 0.25% trypsin non-supplemented with EDTA (Invitrogen, USA), washed and resuspended in PBS. Apoptotic cells were identified by double supravital staining with recombinant FITC-conjugated Annexin V and PI, using the Annexin V/PI-FITC Apoptosis Detection kit (Becton, Dickinson and Company, USA) according to the manufacturer’s recommendations. After 15 min incubation in the dark, the samples were subjected to flow cytometry analysis using BD FACS CantoII flow cytometer (Becton, Dickinson and Company, USA), the data obtained were analyzed by Flow Jo software.

### Protein extraction and western blotting

All protein extraction handling was performed on ice, washed with cold PBS, lysed in proper volume of cell lysis buffer containing protease inhibitor PMSF (1:100, Sigma, Germany), and centrifuged at 12,000*g* at 4 ˚C for 15 min; next, 5 × loading buffer was added to the supernatant. Lysates were boiled for 8 min, then separated by 12% SDS-PAGE at a constant voltage of 70 V, and transferred to polyvinylidene difluoride (PVDF) membranes for 90 min under a constant current of 350 mA. The PVDF membranes were blocked with 5% non-fat milk in TBST containing 0.1% Tween at 37 ˚C for 1 h, and incubated at 4 ˚C overnight with the following primary antibodies: anti-Ubiquitin (#3933, 1:1000, CST, USA), anti-GRP-78(ab229317, 1:1000, Abcam, UK), anti-PERK (#3192, 1:1000, CST, USA), anti-ATF4 (#11815, 1:1000, CST, USA), anti-ATF6 (#65880, 1:1000, CST, USA), anti-CHOP (#2895, 1:1000, CST, USA) and anti-β-actin (#3700, 1:1000, CST, USA). The membranes were washed three times with TBST and then incubated with biotinylated secondary antibodies (1:5000, Fcmacs Biotech, China) for 1 h at 37 ˚C. Eventually, the immunoreactive bands were visualized by FluorChem FC3 chemiluminescence (Protein Simple, USA). Protein contents were densitometrically calculated using the LANE 1D software (Sage, China), and the relative protein expression levels were calculated as target protein/β-actin.

### Measurement of 20S proteasome activity

Activity assays were carried out in a 200 μL reaction volume. Different concentrations of test compounds were added to a black flat/clear bottom 96-well plate containing 1 nM of constitutive 20S proteasome in 50 mM Tris–HCl at pH 7.5 and allowed to sit for 10 min at RT. Fluorogenic substrates were then added and the enzymatic activity measured at 37 °C on a Spectra Max M5e spectrometer by measuring increase in fluorescence unit per minute for 1 h at 380/460 nm. The fluorescence units for the vehicle control were set to 100%, and the ratio of MG132-treated sample set to that of vehicle control was used to calculate the fold change in enzymatic activity.

### Statistical analysis

Values are presented as mean ± SD. All data were analyzed by the SPSS 19.0 statistical software. Comparisons between two groups were performed by the Independent Samples t-test, while comparisons among multiple groups were performed using a one-way analysis of variance (ANOVA) with Tukey’s multiple comparison post hoc test. Differences were considered statistically significant when* P* < 0.05.

## Results

### Apoptosis and Total ubiquitinated proteins expression in BPD model rats

**Fig. 1:**
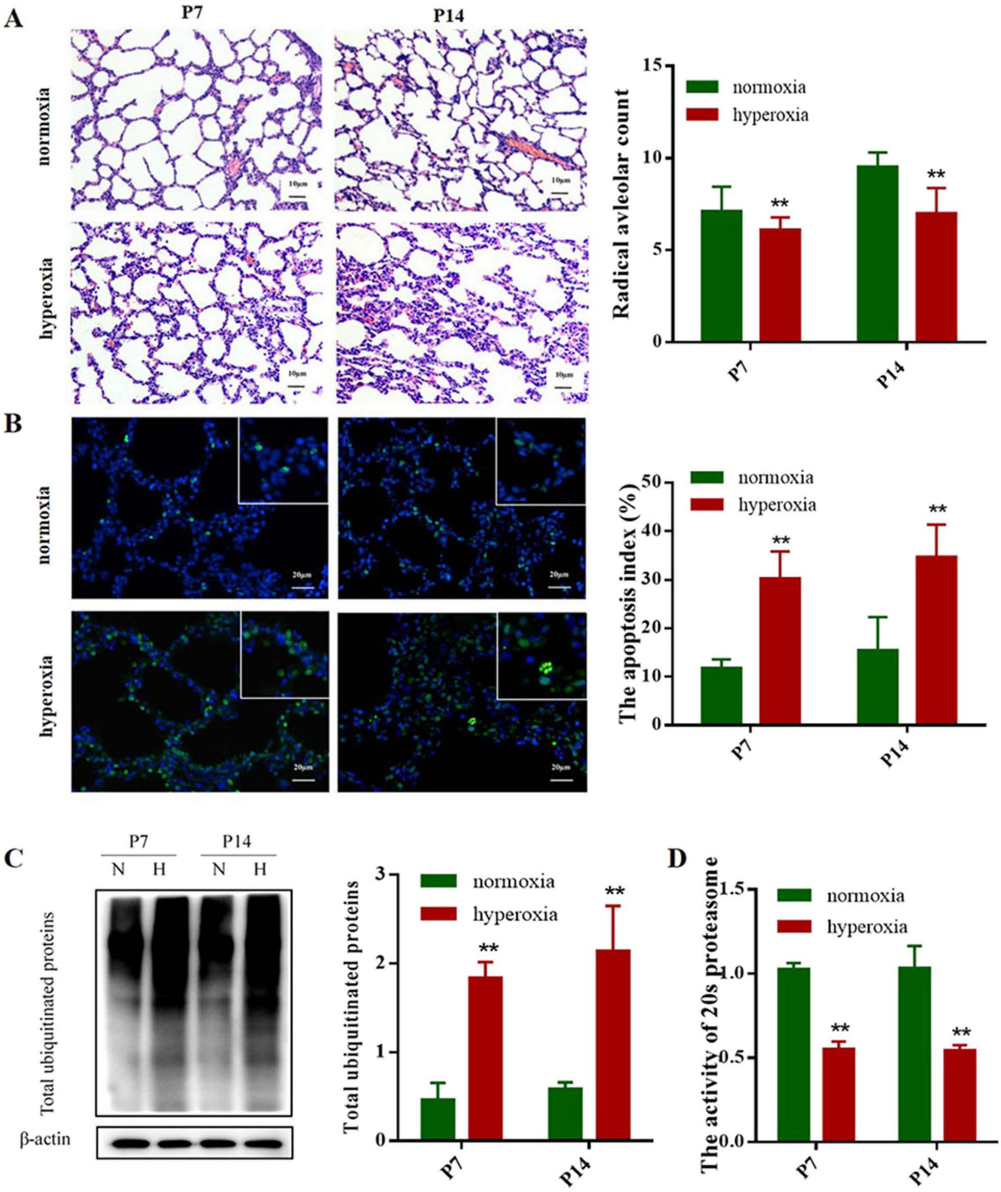
Apoptosis and Total ubiquitinated proteins expression in BPD rats. Neonatal rats were killed at P 7 and P14; normoxia group: 21% O_2,_ hyperoxia group: 80*–*85% O_2_. **A** HE staining assay were used to observe the morphological changes of lung tissue and showed a significant decrease in radial alveolar count (Scale bar = 10 µm; Original magnifications: × 100). **B** TUNEL assay demonstrated a significant increase in the apoptosis index, in a pattern similar to that observed by immunofluorescence staining (Scale bar = 20 µm; Original magnifications: × 200; square frame magnification: × 400). **C** The expression of ubiquitinated proteins were detected by Western blot assays in the four groups and the grouping of blots cropped from the same gel. β-actin was used as the loading control. The part of original data for were presented in Additional file 1: Fig. S1. **D** Reagent kit with specific fluorescent substrate was utilized to measure the activity of 20 s proteasome. Values represent mean ± SD; ** *P* < 0.05 versus normoxia group

Lung histology of the rats exposed to hyperoxia was characterized by decreased septation, distal air space enlargement and a reduction in complexity, which was similar to the histology observed in human infants with BPD [22]. Three newborn rats from different litters were dissected in each group. The survival rate of pups in normoxia and hyperoxia group were both 100%. To quantity the alveolar septation and alveologenesis, Radial alveolar count (RAC) were performed. Compared with the rats exposed to room air, RAC were lower in the rats exposed to hyperoxia (Fig. 1A). These results demonstrated the animal model of BPD was successful. Apoptosis was analyzed using a TUNEL assay following exposure to hyperoxia or room air for P 7 and P 14. As shown by the TUNEL results in Fig. 1B, apoptosis was induced in the lungs of the rats exposed to hyperoxia conditions. A few TUNEL-positive cells were observed in the control group, however, numerous positive cells were observed in the hyperoxia group (Fig. 1B). Under hyperoxia conditions, the levels of total ubiquitinated proteins were significantly increased relative to normal oxygen conditions at both P7 and P14 (Fig. 1C, which is consistent with our results on the apoptosis index in the lung tissue. Further reagent kit with specific fluorescent substrate was utilized to measure the activity of 20 s proteasome and revealed that the level of activity was significantly decreased during hyperoxia conditions (Fig. 1D). These results indicated that ubiquitin or ubiquitin proteasome pathway plays a vital role in apoptosis during the development of BPD.

### Hyperoxia caused AECII apoptosis and ubiquitin proteasome pathway changed

**Fig. 2:**
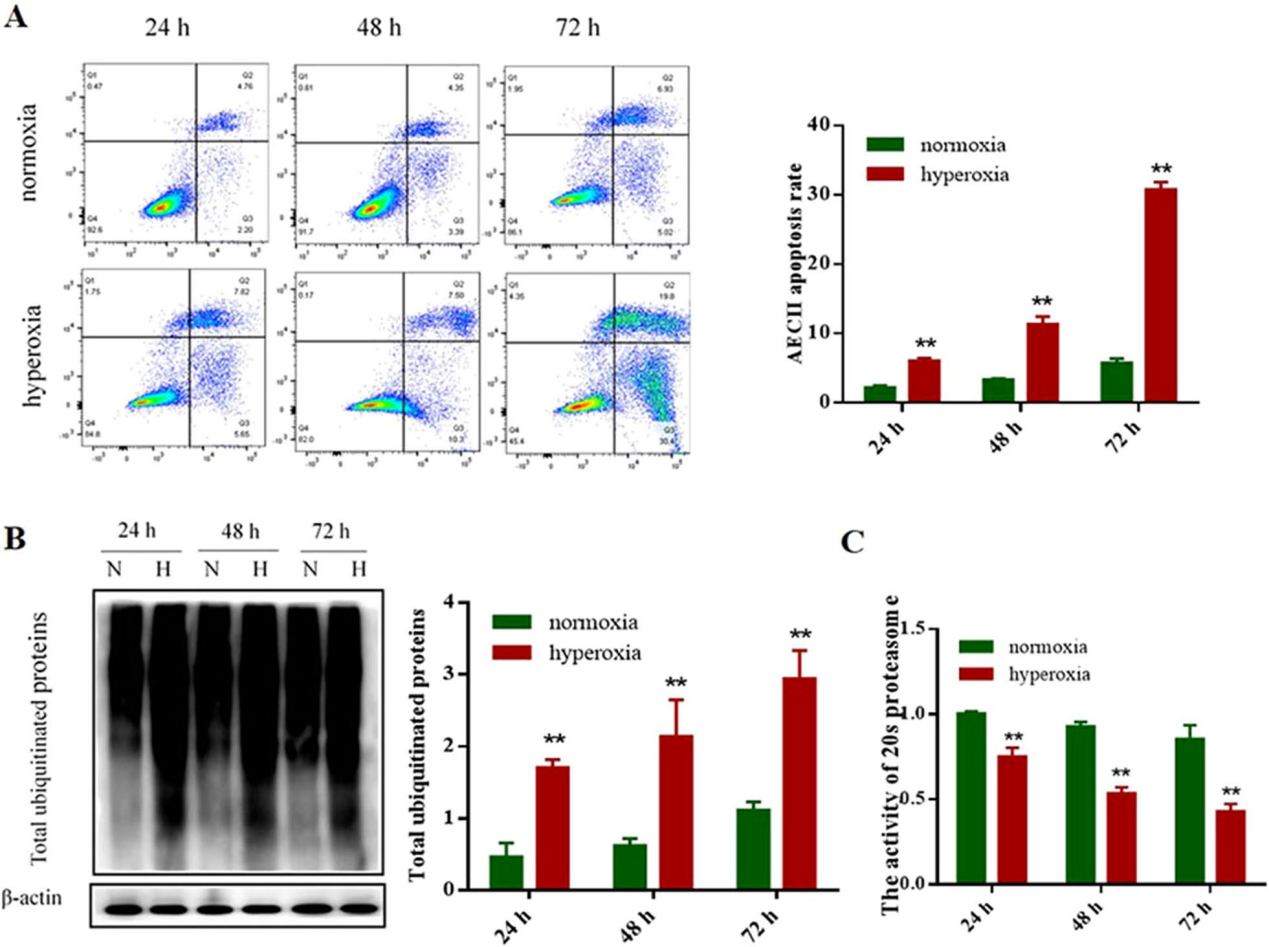
Hyperoxia caused AECII apoptosis and ubiquitin proteasome pathway changed. **A** After 24, 48 and 72 h hyperoxia exposure, AECII apoptosis was analyzed by Annexin V-FITC/PI double staining followed by flow cytometry. **B** The expression of ubiquitinated proteins were detected by Western blot assays in different groups and the grouping of gels cropped from the same gel. β-actin was used as the loading control. The part of original data were presented in Additional file 1: Fig. S2. **C** Reagent kit with specific fluorescent substrate was utilized to measure the activity of 20 s proteasome. Values represent mean ± SD; ** *P* < 0.05 versus normoxia group

To determine whether ubiquitin or ubiquitin proteasome pathway affects BPD development by participating in the apoptosis of AECII, AECII cells were cultured in vitro in the current study and exposed to hyperoxia. As shown by flowcytometry analysis (Annexin V/PI double staining), the apoptosis rate of AECII was low in normoxia group. However, time prolonged exposure to hyperoxia increased number of apoptotic cells and the differences between hyperoxia and normoxia group at 24, 48 and 72 h were statistically significant (*P* < 0.05), pointing out that hyperoxia may induce AECII apoptosis in a time-dependent manner (Fig. 2A). In contrast to the normoxia group, the hyperoxia group showed increased expression of total ubiquitinated proteins after 24, 48 and 72 h exposure to 95% oxygen (Fig. 2B). Compared with it in normoxia group, the level of 20 s proteasome activity was significantly decreased in hyperoxia group (Fig. 2C). Taken together, these results suggested that ubiquitin or ubiquitin proteasome pathway participate in apoptosis of AECII during hyperoxia condition.

### Effects of hyperoxia on GRP-78, CHOP and ERS sensors expression

**Fig. 3:**
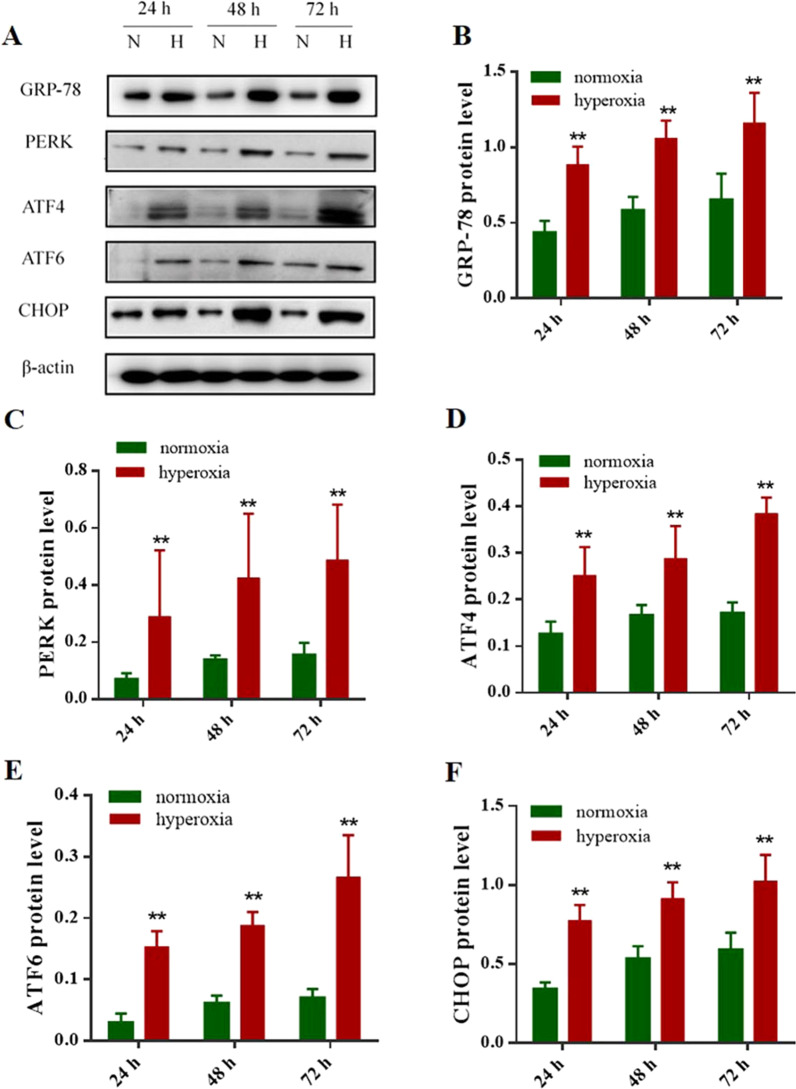
Effects of the protein expression of GRP-78, PERK, ATF4, ATF6 and CHOP in AECII after hyperoxia exposure. **A** Western blot analysis of the AECII that were exposed to 95% oxygen (hyperoxia) or not (normoxia) for 24, 48 and 72 h, 30 g of total protein per well were loaded and subjected to Western blot analysis with corresponding antibodies shown on the left. The part of original data were presented in Additional file 1: Fig. S3. **B**–**F** Quantification of signals on the Fig. 3A, the results showed an increase in GRP78, PERK, ATF4, ATF6 and CHOP protein expression in hyperoxia group in comparison with normoxia group in 24, 48, and 72 h. Values are presented as mean ± SD; ** *P* < 0.05 versus normoxia group

GRP-78 is a sentinel marker of ER stress. To determine the effect of hyperoxia on the expression of the ER chaperone GRP-78, the protein levels were analyzed by Western blotting. The results indicated that the protein expression of GRP-78, was elevated after 24, 48 and 72 h exposure to 95% oxygen in comparison with it in normoxia group (*P* < 0.05) (Fig. 3A, B). Thus, 24 h exposure to hyperoxia was sufficient to activate ER stress in the AECIIs. The protein expression of CHOP was increasing gradually over time in the hyperoxia group compared with it in normoxia group (*P* < 0.05) (Fig. 3A, F), indicating that the exposure of AECIIs to 95% oxygen increased the expression of CHOP, a major pro-apoptotic transcription factor induced by ER stress. PERK and ATF6 were ERS sensors, ATF4 were their downstream-activated proteins that activated CHOP expression according to the previously reported mechanism [23–25]. As shown in Fig. 3A, the PERK, ATF4 and ATF6 proteins were also increased after 24, 48 and 72 h exposure to hyperoxia versus the normoxia group (*P* < 0.05) (Fig. 3A, C, D, E). Combined with the above results in Fig. 2, we found that ERS is activated and 20S proteasome activity of UPP is damaged in AECII after hyperoxia exposure, which blocks the degradation of ubiquitinated proteins and causes accumulation in cells, resulting in increased AECII apoptosis. These results suggested that UPP may participate in the ERS mediated AECII apoptosis under hyperoxia condition.

### Effects of MG132 treatment on ubiquitin proteasome pathway in AECII during hyperoxia exposure

**Fig. 4:**
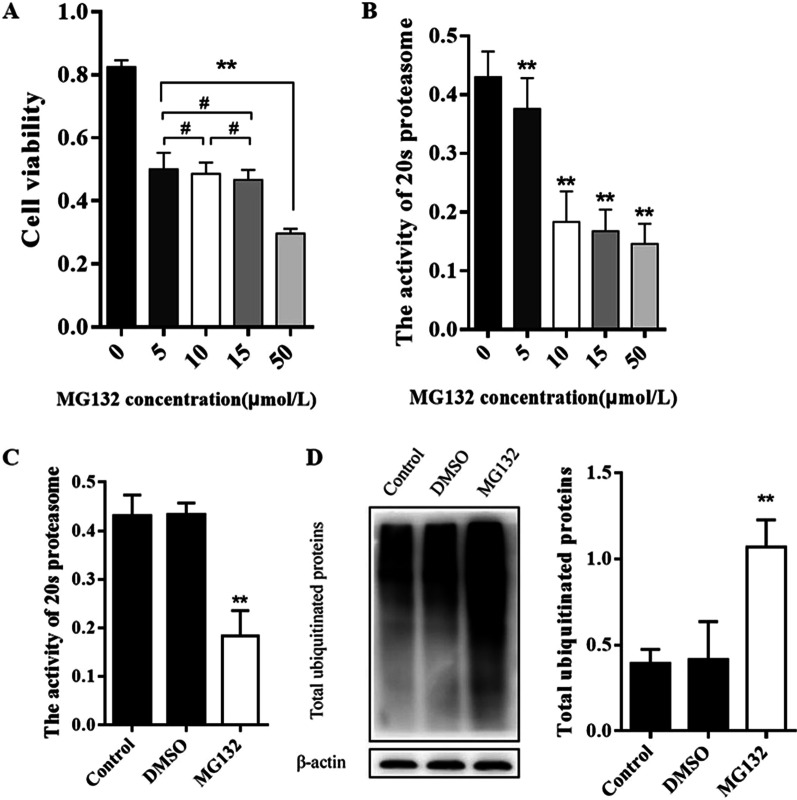
Effects of MG132 treatment on ubiquitin proteasome pathway in AECII during hyperoxia exposure. After MG132 treatment with varying concentration (0, 5, 10, 15 and 50 μmol/L), cell viability was detected by MTT assay (**A**) and the activity of 20 s proteasome was detected by Reagent kit with specific fluorescent substrate (**B**). Compared with Control group and DMSO group, MG132 group (MG132 concentration of 10 μmol/L) showed a decreased activity of 20 s proteasome (**C**) and an increased expression of total ubiquitinated proteins (**D**). The grouping of blots cropped from different parts of the same gel and β-actin was used as the loading control. The part of original data of **D** were presented in Additional file 1: Fig. S4. Values represent mean ± SD; ** *P* < 0.05 versus control

MG132 is a natural peptide aldehyde proteasome inhibitor, which inhibits its activity by covalent binding with the β subunit of 20 s proteasome. AECII cells were treated by MG132 with varying concentration (0, 5, 10, 15 and 50 μmol/L) and cell viability was detected by MTT assay. After MG132 treatment for 72 h cell viability was decreased significantly and continued to show a significant decline with the increasing concentration of MG132 (*P* < 0.05). Compared with no 5 μmol/L MG132 treatment, cell viability were decreased after the 50 μmol/L treatment(*P* < 0.05), while no significant difference varying concentration of 5, 10 and 15 μmol/L(*P* > 0.05) (Fig. 4A). Furthermore, the activity of 20 s proteasome showed a decline with the increasing concentration of MG132 and were significantly decreased after 10 μmol/L MG132 treatment (*P* < 0.05), while slightly decreased after 15 and 50 μmol/L treatment compared with that after 10 μmol/L treatment (Fig. 4B). In view of these results, we finally chose 10 μmol/L as the experimental concentration of MG132 treatment. Compared with hyperoxia group (Control group) and hyperoxia group treated with DMSO (DMSO group), hyperoxia group treated with MG132 (MG132 group) showed a decreased activity of 20 s proteasome and an increased expression of total ubiquitinated proteins (Fig. 4C, D).

### Effects of MG132 treatment on AECII apoptosis and ERS sensors expression during hyperoxia exposure

**Fig. 5:**
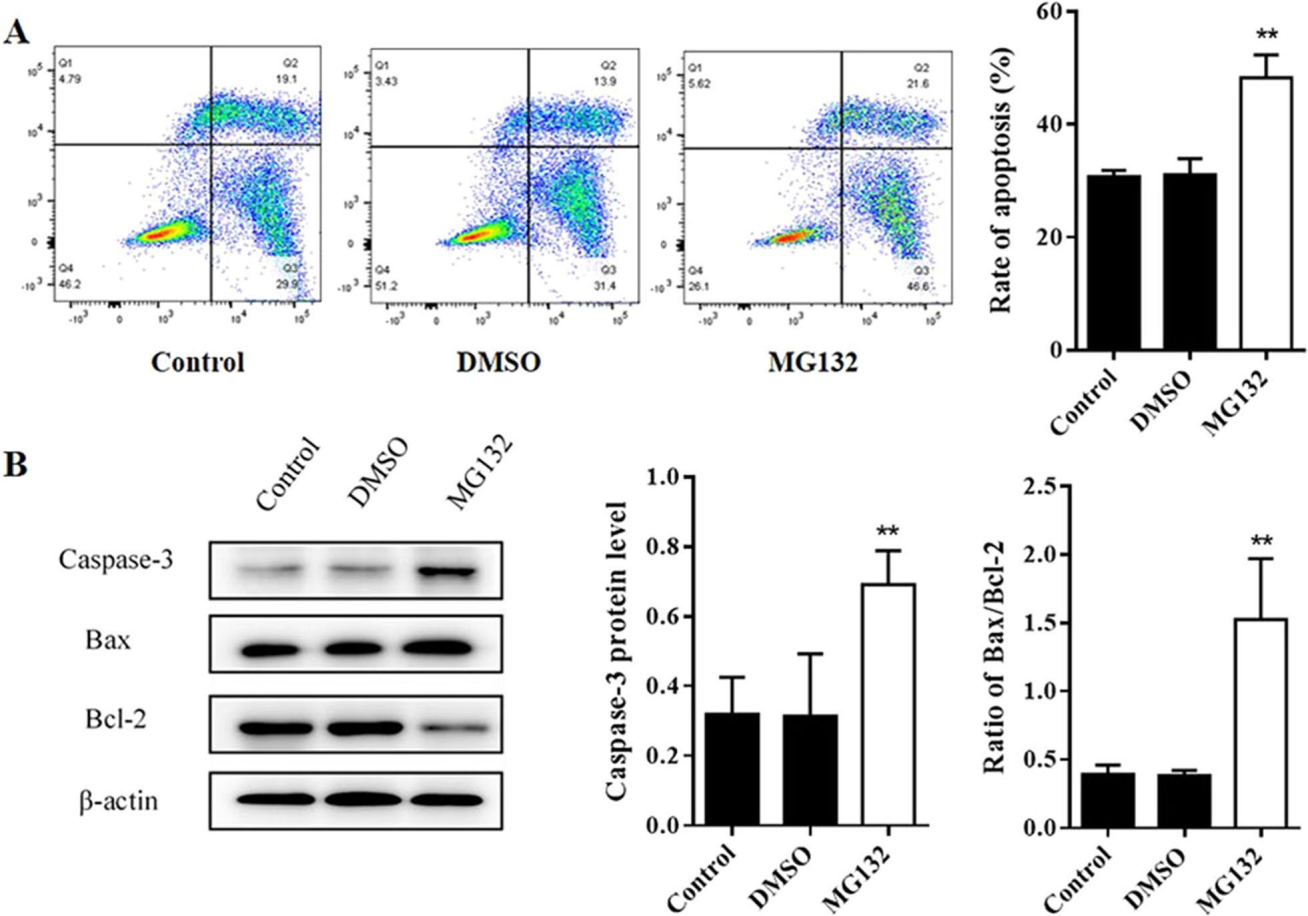
Effects of MG132 treatment on AECII apoptosis during hyperoxia exposure. **a** After MG132 treatment, AECII apoptosis was analyzed by Annexin V-FITC/PI double staining followed by flow cytometry. **b** The proteins expression of Caspases-3, Bax and Bcl-2 were detected by Western blot assays, as well as quantification of Caspases-3 and ratio of Bax/Bcl-2 was measured in different groups. β-actin was used as the loading control. The part of original data were presented in Additional file 1: Fig. S5. Values represent mean ± SD; ** *P* < 0.05 versus control

**Fig. 6:**
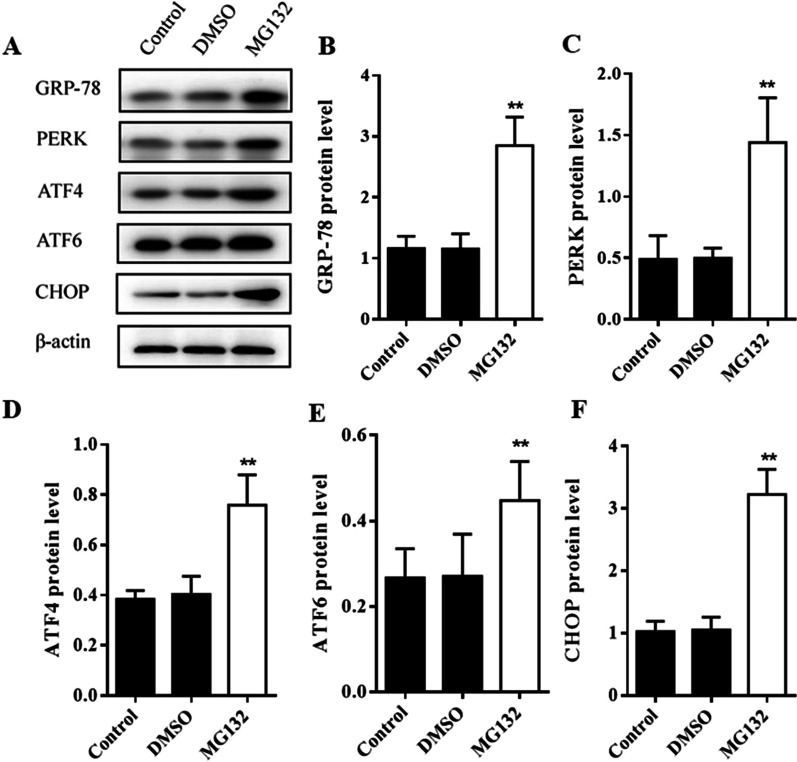
Effects of MG132 treatment on GRP-78, PERK, ATF4, ATF6 and CHOP expression during hyperoxia exposure. **a** Western blot analysis of the AECII that were exposed to hyperoxia for 72 h, 30 g of total protein per well were loaded and subjected to Western blot analysis with corresponding antibodies shown on the left. The part of original data were presented in Additional file 1: Fig. S6. **B**–**F** Quantification of signals on the **A**, the results showed an increase in GRP78, PERK, ATF4, ATF6 and CHOP protein expression in hyperoxia group in comparison with normoxia group in 72 h. Values are presented as mean ± SD; ** *P* < 0.05 versus control

To examine whether MG132 treatment affects AECII apoptosis, flow cytometry analysis and an Annexin V-FITC/PI apoptosis detection kit was used. As shown in Fig. 5A, MG132 treatment promoted apoptosis compared with Control group and DMSO group (*P* < 0.05) (Fig. 5A). In contrast, the level of Caspase-3 protein expression and the ratio of Bax/Bcl-2 were increased after MG132 treatment (*P* < 0.05) (Fig. 5B). These results suggest that the inhibition of 20S proteasome activity leads to AECIIs apoptosis induced by hyperoxia. At the same time, Western blot were performed to analyze protein level of CHOP, ER chaperone GRP-78, as well as ERS sensors PERK, ATF4 and ATF6. As shown in Fig. 6, GRP78, PERK, ATF4, ATF6 and CHOP expression were all increased in varying degree after MG132 treatment(*P* < 0.05) (Fig. 6). Overall, it is valid to consider that MG132 can aggravate the ERS and AECII apoptosis under hyperoxia exposure, and this process is related to UPP.

## Discussion

The characteristic pathological changes of BPD are alveolar dysplasia and pulmonary microvascular remodeling [26]. AECII apoptosis is the initiation of alveolar dysplasia and serves as an important basic event in the neonatal lung injury and development [27, 28]. It may promote the occurrence and development of neonatal BPD. Choo-Wing et al. [29] found that the combined treatment of hyperoxia exposure and interferon gamma on neonatal mice caused ERS to occur in lung tissues, as well as up-regulated the expression of cyclooxygenase-2 (Cox2) and apoptosis of epithelial cells at the same time. Further research showed that CHOP silencing reduces cell apoptosis in lung after hyperoxia mentioned above, suggesting that ERS is involved in hyperoxia induced lung injury.

In the past decade have been increasing evidence supporting that ERS is related to the pathogenesis of pulmonary diseases such as pulmonary fibrosis and asthma [30–32]. Our previous studies found that hyperoxia exposure can increase the expression of GRP-78 and CHOP in lung tissue of premature rats, and is positively correlated with the apoptosis of alveolar epithelial cells [13]. AECII has the function of synthesis, secretion, recycling and removal of surface active substances. AECII serve as alveolar epithelial stem cells, and is closely related to the establishment of gas exchange function and damage repair. Besides, AECII has abundant rough endoplasmic reticulum system, which is suitable for the occurrence of ERS. Konsavage et al. [33] found that AECII and interstitial fibroblasts were the most significant changes of endoplasmic reticulum topology in neonatal rat lung tissue after hyperoxia exposure.

In this study we use hyperoxia-induced BPD rat model and AECII culture experiments to detect the relationship between AECII apoptosis and ERS. As shown by the TUNEL results in BPD rat model, apoptosis was induced in the lungs of the rats exposed to hyperoxia conditions. In combination with previous studies, the level of GRP-78 and CHOP expression were up-regulated in lung tissue of BPD rats [13], we would be correct to assume that hyperoxia activated ER stress in BPD. Further in vitro culture of AECII indicated that the apoptosis rate of AECII in hyperoxia group was higher than it in normoxia group followed by Annexin V/PI double staining, which is consistent with the expression of GRP-78 and CHOP after exposure to hyperoxia. Taken together, the results indicated that ERS participates in hyperoxia-induced AECII apoptosis and it may be a possible molecular mechanisms of alveolar dysplasia in BPD.

As a double-edged sword, ERS can activate UPR to repair protein homeostasis on the one hand and induce apoptosis on the other [34]. The damaged proteins accumulated in the endoplasmic reticulum can be refolded under the guidance of molecular chaperones. Once the folding fails, they are transferred to the degradation pathway, which is mainly performed by UPP. UPP is another major protein degradation system in eukaryotes besides lysosomal pathway. The target protein is ubiquitinated through a series of enzymatic reactions and specifically recognized and degraded by 26 s proteasome [17]. The dysfunction of UPP is related to the pathogenesis of some human diseases. For example, in neurodegenerative diseases such as Alzheimer's disease (AD), the abnormal degradation of tau protein is closely related to the dysfunction of 26 s proteasome [35]. Inhibition of proteasome activity in tumor cells can induce or aggravate ers and promote apoptosis of tumor cells.

As shown in our results, the activity of 20 s proteasome was decreased and the expression of total ubiquitinated proteins after hyperoxia exposure. Furthermore, we observed that the changes in the levels AECII apoptosis is positively correlated with the expression of total ubiquitinated proteins, but negatively correlated with the activity of 20 s proteasome. 20 s proteasome is the core particle with enzymolysis activity in 26 s proteasome. It is known that the decrease of proteasome activity leads to the decrease of ubiquitination protein degradation, and accumulation in cells [36, 37]. This process causes pressure on the endoplasmic reticulum and affects the normal operation of the endoplasmic reticulum. ERS activation mobilized UPP, which the damage of UPP aggravated ERS. They form a vicious circle, and eventually apoptosis occurs when the cells are overloaded.

UPP is another major protein degradation system in eukaryotes besides lysosomal pathway. The target protein is ubiquitinated through a series of enzymatic reactions and specifically recognized and degraded by 26 s proteasome [17, 38]. The dysfunction of UPP is related to the pathogenesis of some human diseases. For example, in neurodegenerative diseases such as AD, the abnormal degradation of tau protein is closely related to the dysfunction of 26S proteasome. Inhibition of proteasome activity in tumor cells can induce or aggravate ERS and promote apoptosis of tumor cells.

In recent years, some proteasome inhibitors have been widely used in cancer research [39, 40]. In human breast cancer cell MCF7, MG132 treatment up-regulated the expression of GRP-78, CHOP, Bax and Caspase-3, and down-regulated the expression of Bcl-2, resulting in increased apoptosis [41]. Our results showed that MG132 treatment promoted AECII apoptosis compared with Control group and DMSO group, suggesting that cell apoptosis increases when proteasome activity is inhibited. Correspondingly, MG132 treatment up-regulated the expression of Caspase-3 and the ratio of Bax/Bcl-2, as well as the expression of ERS related GRP-78, PERK, ATF4, ATF6 and CHOP. It is reasonable to believe that MG132 treatment aggravates the apoptosis of AECII, which is related to the occurrence of ERS. Lawson et al. [42] showed that ERS is not enough to induce pulmonary fibrosis, but it can increase the sensitivity of AECII to injury. Similarly, we speculated that ERS may increase the sensitivity of AECII during lung injury in BPD. On the basis of ERS induced by hyperoxia exposure, the inhibition of proteasome activity seriously affected UPP function, further resulted in the accumulation of injury proteins, up-regulated ERS level and AECII apoptosis.

Our studies shed new insight into the role of UPP in AECII apoptosis of BPD. We provided reliable evidence about the correlation of ERS and AECII apoptosis, and demonstrated that UPP participate in ER stress-induced AECII apoptosis as we observed increased expression of GRP-78 and ERS sensors after MG132 treatment. Although our research provides a possible mechanism that hyperoxia enhance ER stress-induced AECII apoptosis, which decreases the activity of 20 s proteasome and degradation of total ubiquitinated proteins, further studies are required to clarify initiation and progression of AECIIs apoptosis in BPD.

## Conclusions

In conclusion, our findings indicate that UPP may participate in the ERS-induced AECII apoptosis under hyperoxia condition, which is essential for the development of BPD.

## Supplementary Information

**Additional file 1:** The original gels and Institutional Review Board Statement.

## Data Availability

The data presented in this study are available on request from the corresponding author. The data are not publicly available due to privacy.

## Abbreviations

BPD: Bronchopulmonary dysplasia
UPP: Ubiquitin proteasome pathway
ERS: Endoplasmic reticulum stress (ER stress)
SP: Pulmonary surfactant
UPR: Unfolded protein response
eIF2α: Eukaryotic translation initiation factor 2 subunitα
ERAD: ER associated degradation
RACs: Radial alveolar counts
PVDF: Polyvinylidene difluoride
ROP: Pneumonia and retinopathy of prematurity
ROS: Reactive oxygen species
AD: Alzheimer's disease
