# Supplementary material for: The function role of ubiquitin proteasome pathway in the ER stress-induced AECII apoptosis during hyperoxia exposure
Source: BMC Pulm Med. 2021 Nov 22;21:379. doi: 10.1186/s12890-021-01751-9 (PMC8607682; doi:10.1186/s12890-021-01751-9)
Supplement: Supplementary file 1 — Additional file 1: The original gels and Institutional Review Board Statement. [file 12890_2021_1751_MOESM1_ESM.docx]

The function role of ubiquitin proteasome pathway in the ER stress-induced AECII apoptosis during hyperoxia exposure

Yue Zhu^†^, Huimin Ju^†^, Hongyan Lu* , Wei Tang, Junying Lu and Qiuxia Wang

Department of Pediatrics, Affiliated Hospital of Jiangsu University, Zhenjiang, Jiangsu 212000, P.R.China

^†^ These authors contributed equally to this work

* Correspondence: lhy5154@163.com; Tel.: +86-0511-8508-2260

**Supplementary information**

**Supplementary Materials:**

**Supplementary Figure S1** Uncropped Western blots for Figure 1C.

**Supplementary Figure S2** Uncropped Western blots for Figure 2B.

**Supplementary Figure S3** Uncropped Western blots for Figure 3A.

**Supplementary Figure S4** Uncropped Western blots for Figure 4D.

**Supplementary Figure S5** Uncropped Western blots for Figure 5B.

**Supplementary Figure S6** Uncropped Western blots for Figure 6A.

**Supplementary Figure S7** Institutional Review Board Statement.

**Supplementary Figure S8** Uncropped Western blots for CHOP, GRP-78, PERK, ATF4 and ATF6


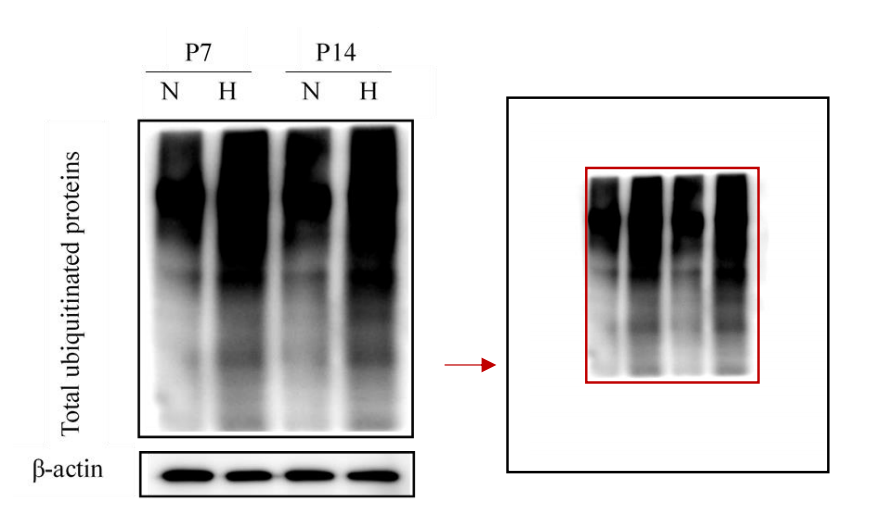


**Supplementary Figure S1** Uncropped Western blots for Figure 1C


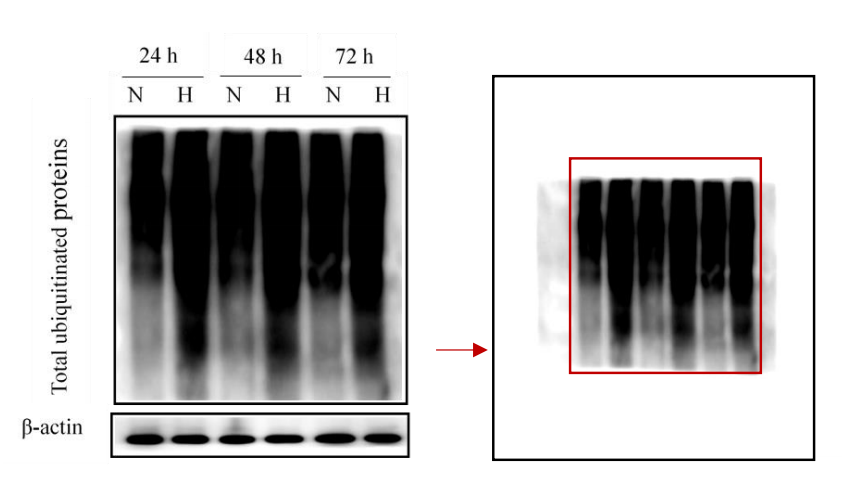


**Supplementary Figure S2** Uncropped Western blots for Figure 2B


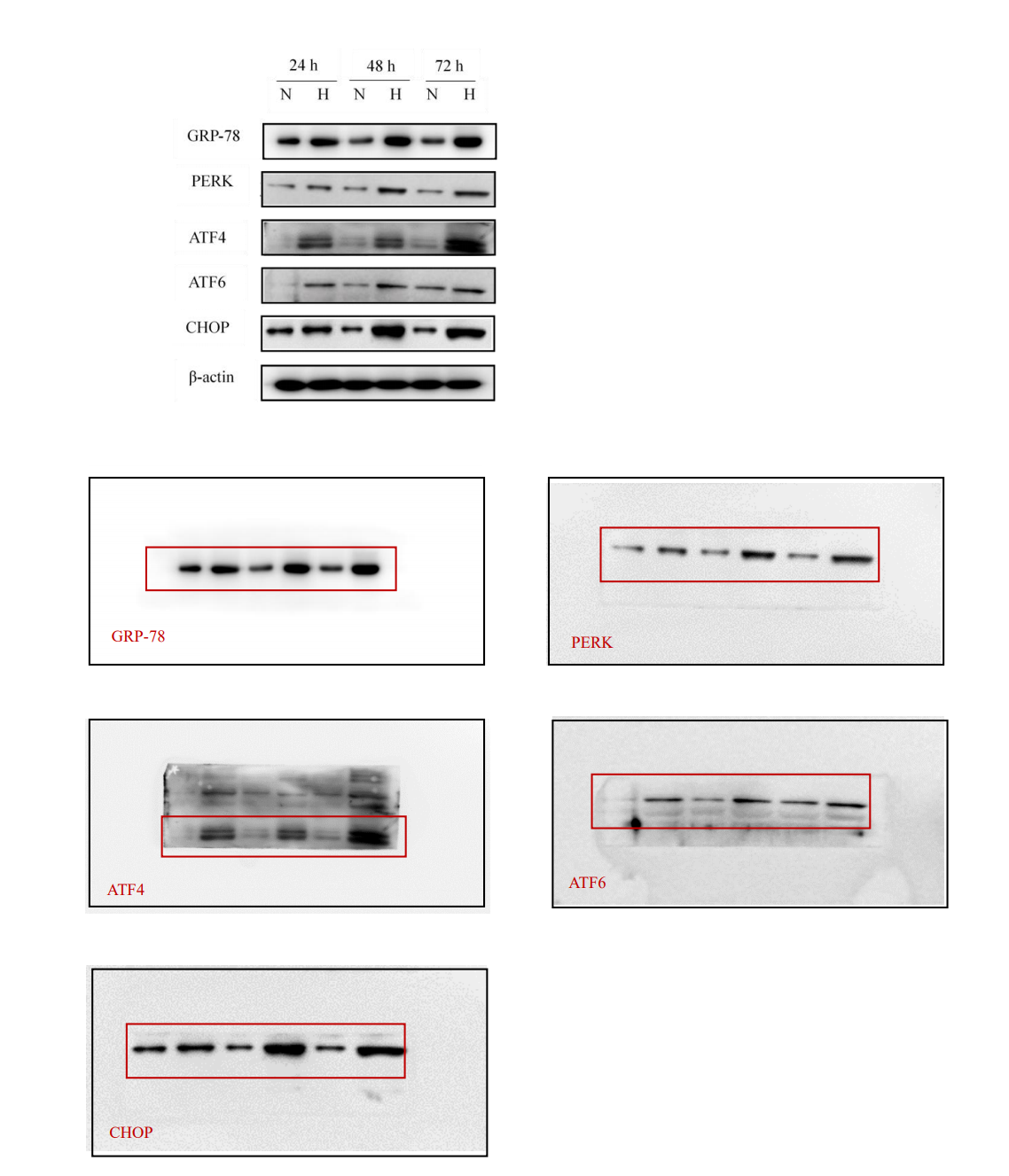


**Supplementary Figure S3** Uncropped Western blots for Figure 3A


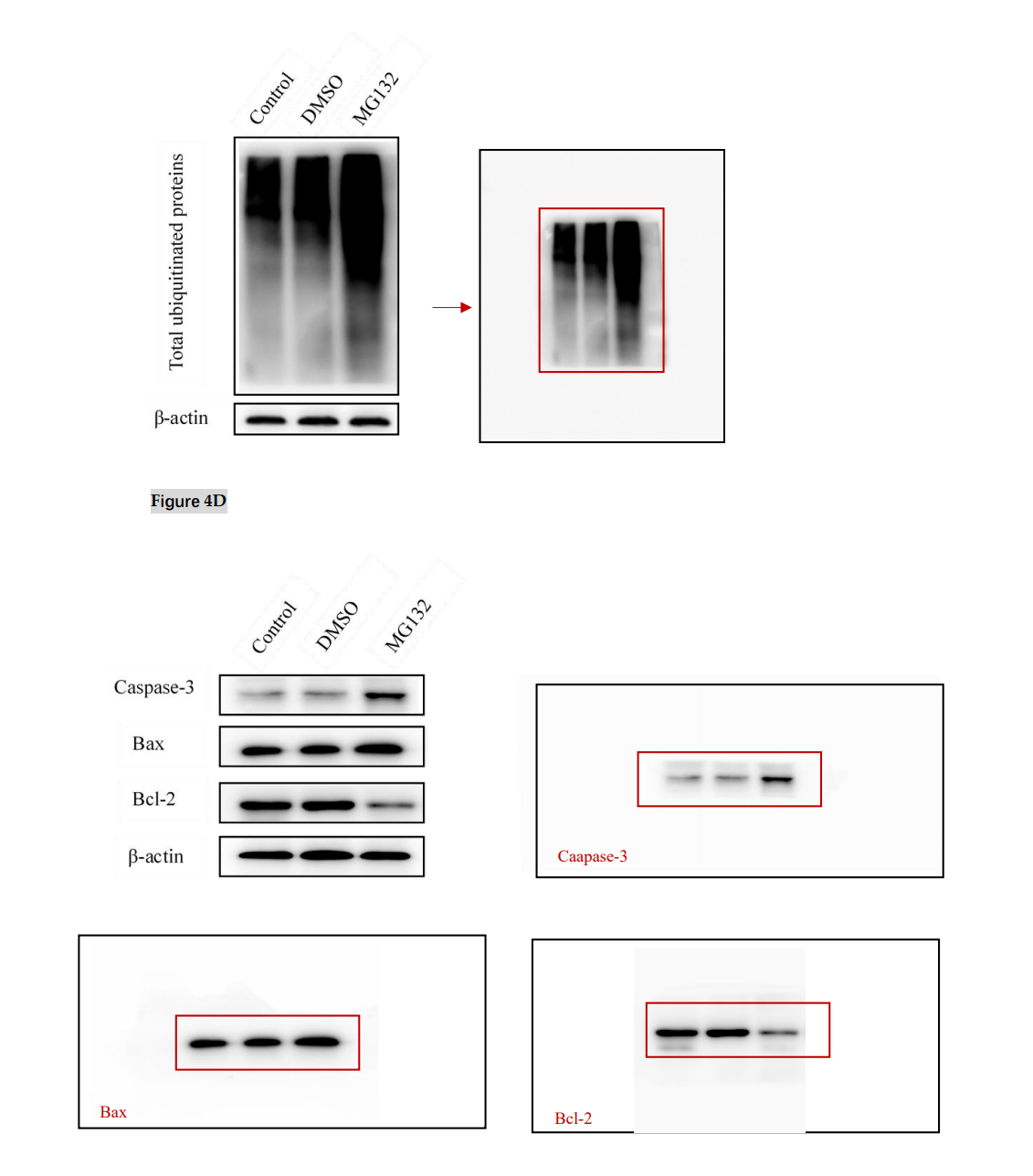


**Supplementary Figure S4** Uncropped Western blots for Figure 4D


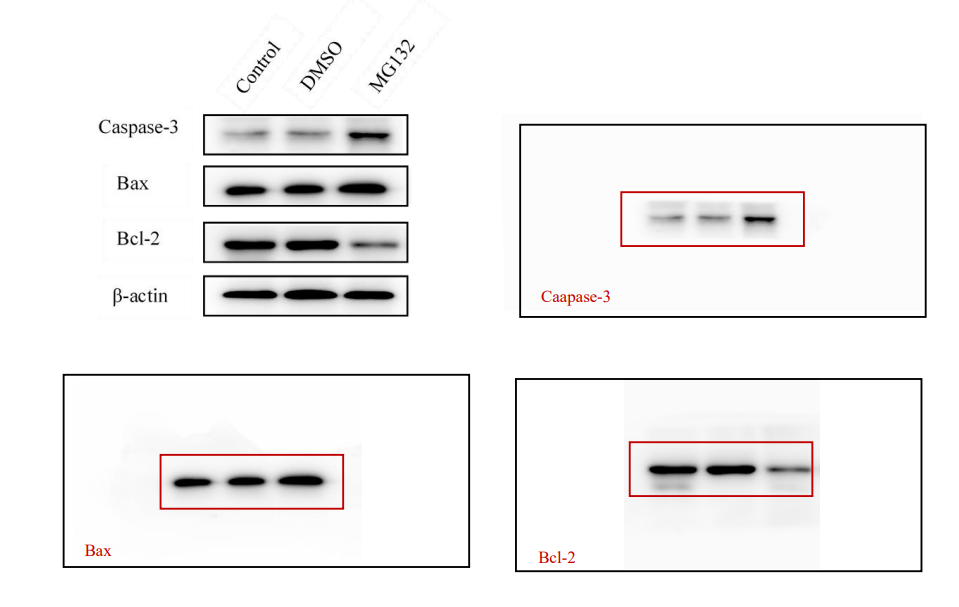


**Supplementary Figure S5** Uncropped Western blots for Figure 5B


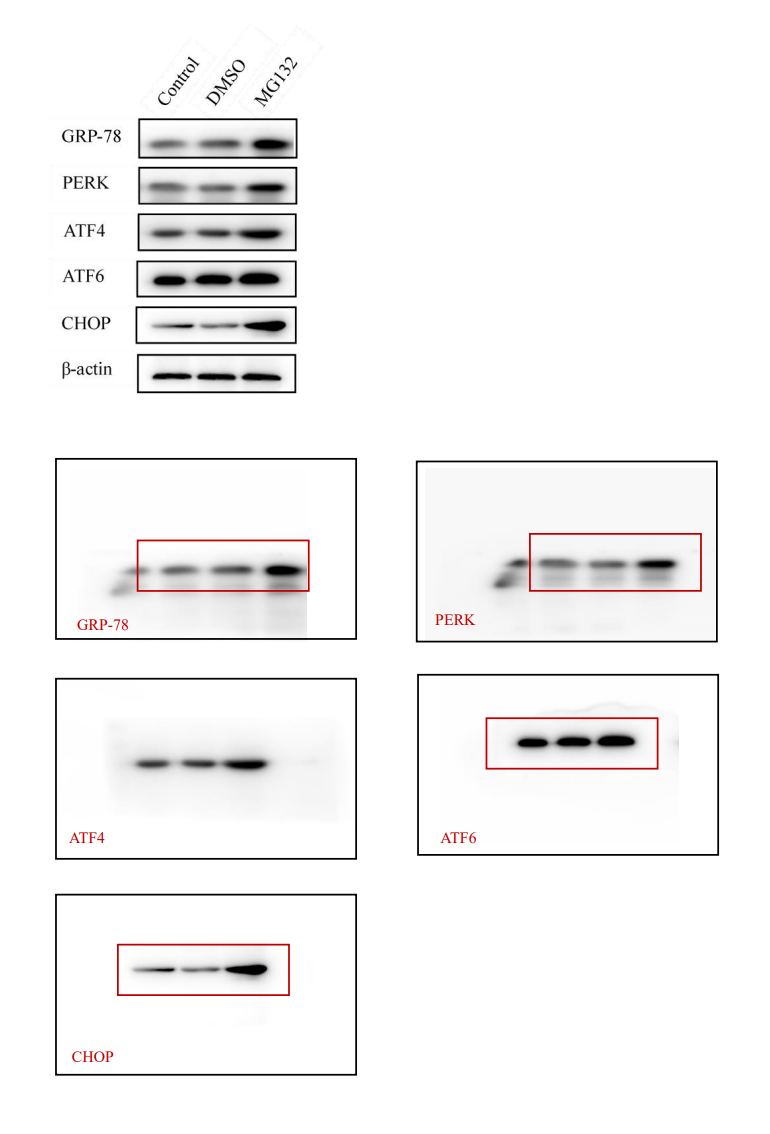


**Supplementary Figure S6** Uncropped Western blots for Figure 6A


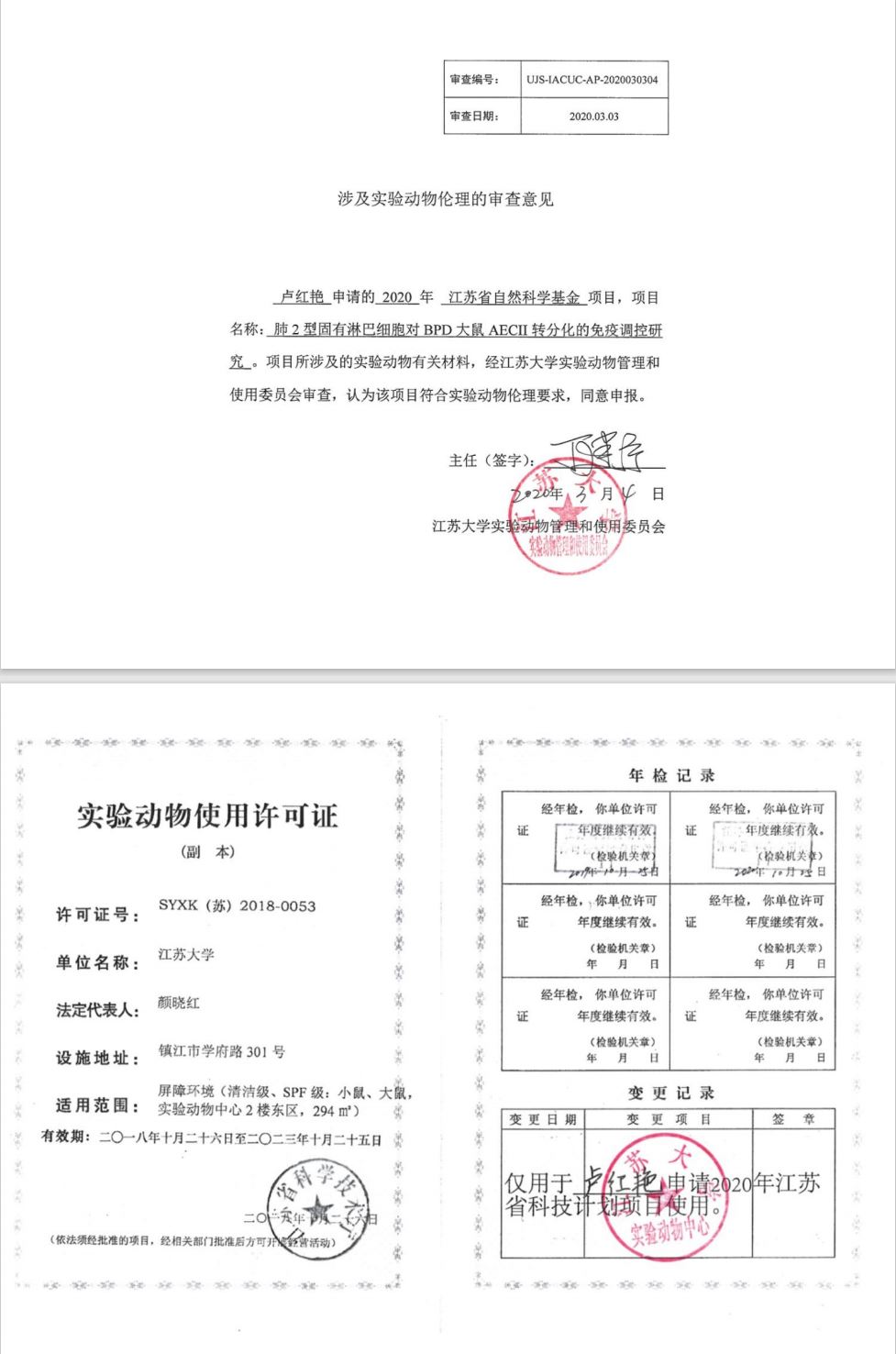


**Supplementary Figure S7** Institutional Review Board Statement. The study was reported in accordance with ARRIVE guidelines and approved by the Animal Center at Jiangsu University (protocol No. UJS-IACUC-AP-2020030304 and approved on 2020.03.03)


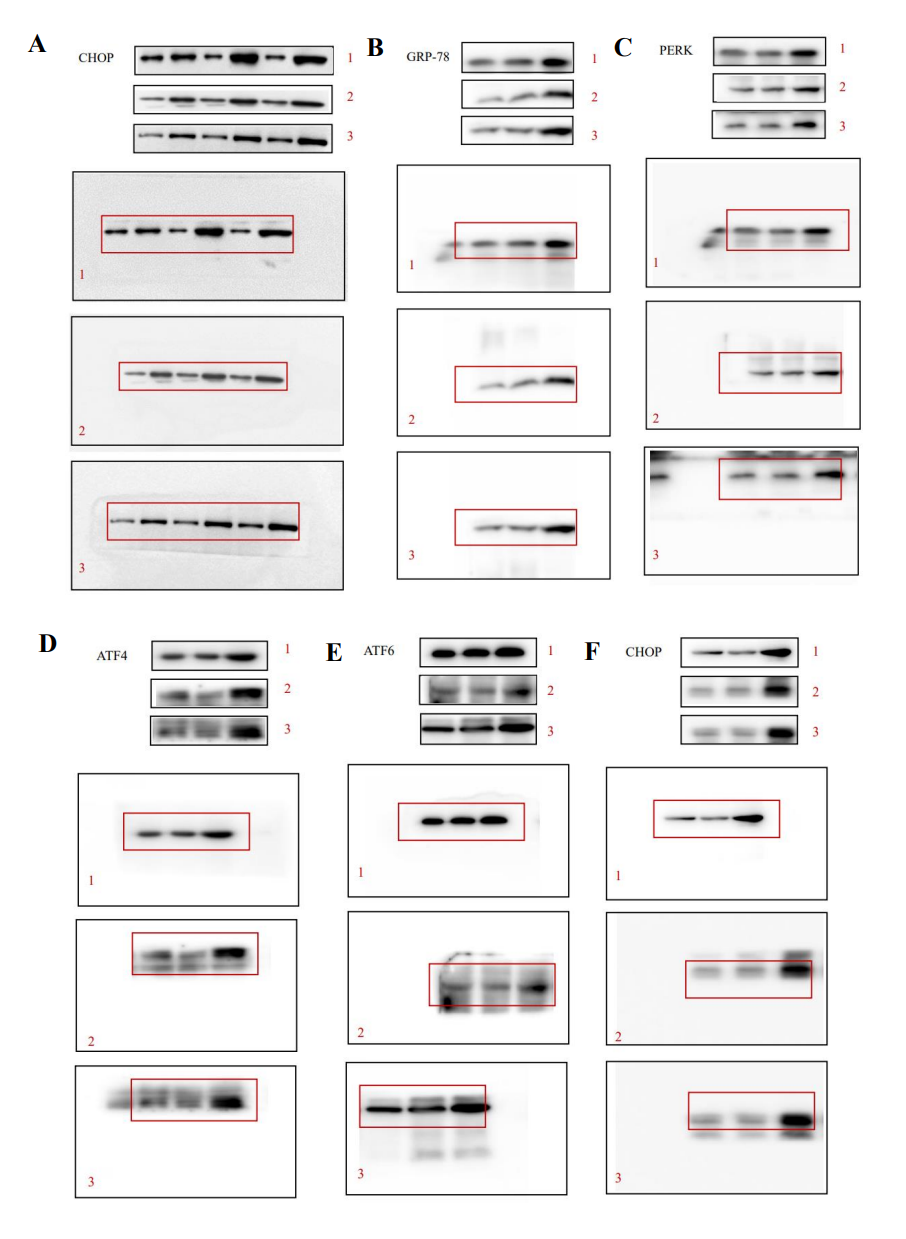


**Supplementary Figure S8** Uncropped Western blots for CHOP, GRP-78, PERK, ATF4 and ATF6, which mentioned in “Point-by-point response”.
